# Nationwide real-world implementation of AI for cancer detection in population-based mammography screening

**DOI:** 10.1038/s41591-024-03408-6

**Published:** 2025-01-07

**Authors:** Nora Eisemann, Stefan Bunk, Trasias Mukama, Hannah Baltus, Susanne A. Elsner, Timo Gomille, Gerold Hecht, Sylvia Heywang-Köbrunner, Regine Rathmann, Katja Siegmann-Luz, Thilo Töllner, Toni Werner Vomweg, Christian Leibig, Alexander Katalinic

**Affiliations:** 1 Institute for Social Medicine and Epidemiology, University of Lübeck, Lubeck, Germany; 2 Vara, Berlin, Germany; 3 Diagnosticum Visiorad, Pinneberg, Germany; 4 Reference Center Mammography North, German Breast Cancer Screening Program, Oldenburg, Germany; 5 Reference Center Mammography Munich, German Breast Cancer Screening Program and FFB gGmbH, Munich, Germany; 6 Radiology Center Schwarzer Bär, Hannover, Germany; 7 Reference Center Mammography Berlin, German Breast Cancer Screening Program, Berlin, Germany; 8 Clinic Dr. Hancken, Stade, Germany; 9 Radiological Institute Dr. von Essen, Koblenz, Germany

**Keywords:** Breast cancer, Cancer screening, Machine learning

## Abstract

Artificial intelligence (AI) in mammography screening has shown promise in retrospective evaluations, but few prospective studies exist. PRAIM is an observational, multicenter, real-world, noninferiority, implementation study comparing the performance of AI-supported double reading to standard double reading (without AI) among women (50–69 years old) undergoing organized mammography screening at 12 sites in Germany. Radiologists in this study voluntarily chose whether to use the AI system. From July 2021 to February 2023, a total of 463,094 women were screened (260,739 with AI support) by 119 radiologists. Radiologists in the AI-supported screening group achieved a breast cancer detection rate of 6.7 per 1,000, which was 17.6% (95% confidence interval: +5.7%, +30.8%) higher than and statistically superior to the rate (5.7 per 1,000) achieved in the control group. The recall rate in the AI group was 37.4 per 1,000, which was lower than and noninferior to that (38.3 per 1,000) in the control group (percentage difference: −2.5% (−6.5%, +1.7%)). The positive predictive value (PPV) of recall was 17.9% in the AI group compared to 14.9% in the control group. The PPV of biopsy was 64.5% in the AI group versus 59.2% in the control group. Compared to standard double reading, AI-supported double reading was associated with a higher breast cancer detection rate without negatively affecting the recall rate, strongly indicating that AI can improve mammography screening metrics.

## Abstract (web summary)

In a large-scale prospective study run across 12 sites in Germany and involving 463,094 women and 119 radiologists, AI-supported screening increased breast cancer detection rates by a significant margin without affecting the recall rate.

## Main

Mammography screening programs contribute to reducing mortality associated with breast cancer^1,2^. However, there is still room for improvement in breast cancer screening. On the one hand, the sensitivity of screening could be improved to lead to lower interval cancer rates and more effective treatment of patients with breast cancer. On the other hand, the specificity of screening could be increased to reduce the recall rate by minimizing false-positive results, which can cause anxiety and uncertainty among the screened women. Thus, a higher specificity would lessen the burden on the screening participants and the healthcare system by minimizing unnecessary, invasive and costly medical procedures.

Furthermore, the programs generate substantial volumes of mammograms, which, in many programs (including the German mammography screening program), require interpretation by two independent radiologists; a consensus conference or arbitration may also be necessary to achieve high sensitivity and specificity^3,4^. Thus, the radiologists’ work involves the repetitive task of interpreting hundreds of images per week, most of which have no signs of breast cancer. This approach heavily relies on human expertise, yet screening programs are experiencing a lack of radiologists^5^. With national and international guidelines recently recommending mammography screening also for the age groups 40/45–49 and 70–74 years, the workload is expected to increase^6–8^.

Integrating artificial intelligence (AI) into breast cancer screening workflows could alleviate some of the problems that screening programs face. Retrospective studies have shown that AI has comparable and sometimes superior accuracy to radiologists, suggesting that the technology can support radiologists in interpreting mammograms by improving the identification of subtle abnormalities that might otherwise elude human readers and by reducing the reading workload^9–11^. Growing evidence indicates that AI detects 20–40% of interval cancers that can retrospectively be seen or suspected on prior screening mammograms but were missed by radiologists^12–14^. Using retrospective data in studies evaluating the impact of AI on screening metrics is limited by the uncertainty of outcomes in women whose mammography findings were flagged as suspicious by AI only and were, therefore, not referred for consensus conferences or further assessments. A growing body of prospective evidence demonstrates the potential of AI to improve screening metrics and additionally reduce reading workload. The MASAI (MAmmography Screening with AI) trial^15^, the ScreenTrustCAD study^16^ and a study by Ng et al.^17^ all reported increased cancer detection for workflows incorporating AI, but the results on recall rates were inconsistent. However, these studies are limited by small sample sizes (which restrict the analysis of subgroups) and by the lack of heterogeneity in terms of screening sites, mammography equipment vendors and the radiologists involved, thereby reducing their generalizability to real-world settings.

In a retrospective analysis, Leibig et al.^18^ demonstrated that the use of AI in a decision referral approach, in which AI confidently predicts normal or highly suspicious examination results and refers uncertain results to the radiologists’ expertise, yielded superior metrics than AI or radiologists alone. In the PRAIM (PRospective multicenter observational study of an integrated AI system with live Monitoring) implementation study embedded in the German mammography screening program, we investigated whether the performance metrics achieved by double reading using an AI-supported CE (Conformité Européenne)-certified medical device with a decision referral approach were noninferior to those achieved by double reading without AI support in a real-world setting. Here, we report the impact of AI on cancer detection and recall rates.

## Results

**Fig. 1:**
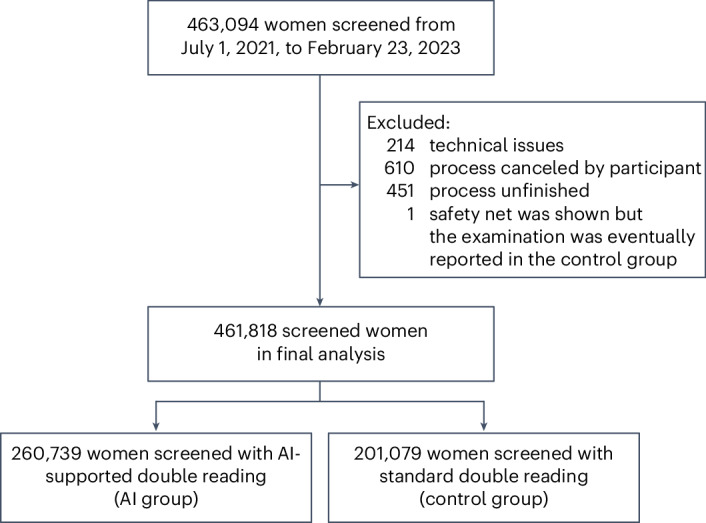
Study profile. The flowchart shows the inclusion of study participants and their assignment into groups.

The study was conducted within Germany’s organized breast cancer screening program targeting asymptomatic women aged 50–69 years (Fig. 1). All women participating in the screening program were eligible for study inclusion. Between July 1, 2021, and February 23, 2023, data from screening participants were collected from 12 screening sites that used the AI system (Extended Data Table 1). In the German mammography screening program, which is based on a binding national guideline, four two-dimensional mammograms (craniocaudal and mediolateral oblique views of each breast) are taken for each participating woman. These mammograms are initially read independently by two radiologists (sometimes, a third radiologist supervises). If at least one radiologist deems the case suspicious, a consensus conference is held. The participants of the consensus conference are at least the two initial readers and one head radiologist, but more radiologists of the screening site can participate. If the suspicious finding persists in the consensus conference, the woman is recalled for further diagnostic assessments, which can include, among others, ultrasonography, digital breast tomosynthesis, magnification views, contrast-enhanced mammography or magnetic resonance imaging.

For the study, examinations were assigned to the AI group when at least one of the two radiologists read and submitted the report with the AI-supported viewer. All examinations for which neither radiologist submitted the report using the AI-supported viewer formed the control group. The study group assignment was unknown to the women and radiographers as it was not yet assigned at the time of image acquisition. After image acquisition, AI predictions were computed for all women but were displayed only to radiologists using the AI-supported viewer. The radiologists performing the first and second reads were free to use either their existing reporting and viewer software without AI support or the AI-supported viewer. The decision to use the AI-supported viewer was made on a per-examination basis (that is, one radiologist typically delivered examinations for both the AI and control groups). Radiologists in a reader set independently chose whether to use the AI-supported viewer. The AI results were not disclosed to the other radiologist if they did not also choose to use the AI viewer.

**Extended Data Fig. 1:**
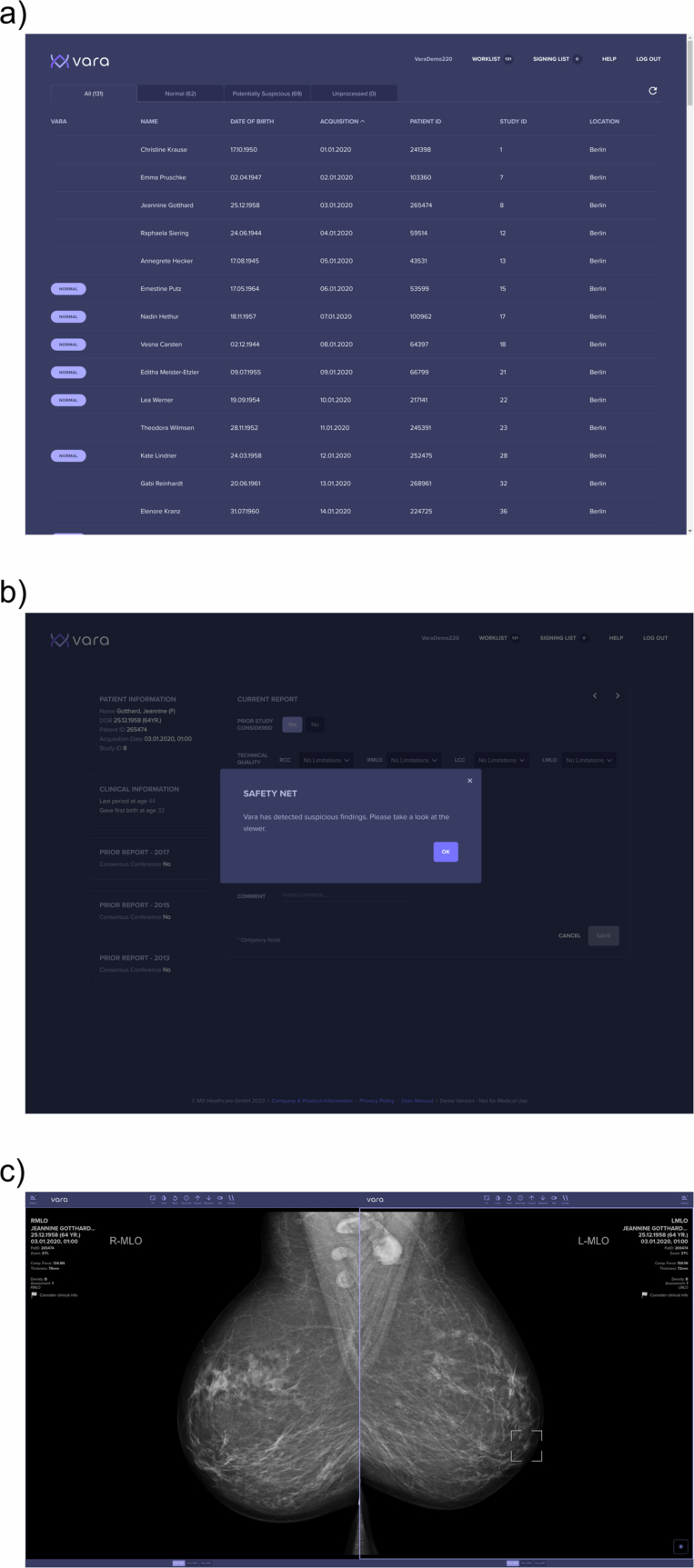
AI-supported Viewer Screenshots. **a**) The screenshot shows a worklist with subset of examinations tagged as normal. Radiologists can also choose to read only normal examinations or only not normal examinations (‘potentially suspicious’). Names and dates of birth are not real and were sampled randomly from a list of common first and last names. **b**) and **c)** When radiologists assess a case as normal (BI-RADS 1 or 2) but the safety net triggered, an alert is shown (**b**) and a suspicious region is highlighted in the viewer (**c**), asking the radiologists to reconsider.

The AI system used was Vara MG (from the German company Vara), a CE-certified medical device designed to display mammograms (viewer software) and preclassify screening examinations to assist radiologists in their reporting routine. The performance of previous versions of the AI software has been previously reported^12,18^. When using the AI-supported viewer, radiologists were supported by two AI-based features (Extended Data Fig. 1):

1. Normal triaging. The software selects a subset of all examinations deemed highly unsuspicious by the AI model. These examinations are tagged ‘normal’ in the worklist.
2. Safety net. The software selects a subset of all examinations deemed highly suspicious by the AI model. Radiologists first read the screening examination without any further AI support. When the radiologists interpret an examination as unsuspicious, the safety net is activated with an alert and a suggested localization of the suspicious region(s) in the images. The radiologists are then prompted to review their decision and either accept or reject the safety net’s suggestion.

### Characteristics of the study population

Overall, 461,818 women who attended mammography screening at the 12 screening sites participated in the study. A total of 119 radiologists constituting 547 reader sets interpreted the examinations. Mammography hardware systems from five different vendors were used (Extended Data Table 2). Of all the participating women, 260,739 were screened in the AI group (with the AI-supported viewer being used by only one reader for 152,970 women and by both readers for 107,769 women) and 201,079 were screened in the control group. Table 1 presents the characteristics of the screened women and the detected breast cancers by study group. Of the screened women, 41.9 per 1,000 had suspicious findings and were recalled for further assessment. A quarter of them (10.4 per 1,000) underwent biopsy procedures, and 6.2 per 1,000 were finally diagnosed with breast cancer. Most (79.4%) of the cancers were classified as invasive, and 18.9% were ductal carcinoma in situ (DCIS).

**Table 1:** Characteristics of the study population overall and by study group

Characteristics	AI group (*n* = 260,739)	Control group (*n* = 201,079)	Overall (*n* = 461,818)
Age (years)			
Median (IQR)	58 (54–63)	58 (54–63)	58 (54–63)
Missing, *n* (%)	2 (<0.1%)	0 (0%)	2 (<0.1%)
50–59 years, *n* (%)	114,365 (43.9%)	87,781 (43.7%)	202,146 (43.8%)
Breast density, *n* (%)			
Nondense	165,332 (63.4%)	137,889 (68.6%)	303,221 (65.7%)
Dense	94,955 (36.4%)	63,125 (31.4%)	158,080 (34.2%)
Missing	452 (0.2%)	65 (<0.1%)	517 (0.1%)
Screening round, *n* (%)			
First	43,449 (16.7%)	35,680 (17.7%)	79,129 (17.1%)
Follow-up	217,290 (83.3%)	165,399 (82.3%)	382,689 (82.9%)
Screening sites			
Number of sites	11	12	12
Screened women per unit, median (IQR)	18,503 (15,703–29,466)	13,341 (4,860–21,980)	40,101 (27,060–47,858)
Radiologists			
Number	110	117	119
Number of reader sets	387	485	547
Screened women per reader set, mean (s.d.)	477 (1,069)	368 (637)	844 (1,326)
Minimum/maximum	0/8,664	0/5,940	1/8,939
Consensus conference			
Consensus conferences, *n* (%)	32,341 (12.4%)	21,996 (10.9%)	54,337 (11.8%)
Recall			
Recalls, *n* (per 1,000)	11,603 (44.5)	7,727 (38.4)	19,330 (41.9)
PPV of recall (%)	14.5	15.6	14.9
Preoperative biopsy			
Biopsy recommended, *n* (per 1,000)	2,843 (10.9)	1,981 (9.9)	4,824 (10.4)
PPV of biopsy (%)	59.0	60.5	59.6
Detected breast cancers			
Breast cancers, *n* (per 1,000)	1,679 (6.4)	1,202 (6.0)	2,881 (6.2)
Invasiveness, *n* (% of cancer detected)			
DCIS	341 (20.3%)	203 (16.9%)	544 (18.9%)
Invasive	1,308 (77.9%)	981 (81.6%)	2,289 (79.4%)
Other	30 (1.8%)	18 (1.5%)	48 (1.7%)
Breast cancer stage (UICC), *n* (% of cancer detected)			
0	341 (20.3%)	203 (16.9%)	544 (18.9%)
1	881 (52.5%)	588 (48.9%)	1,469 (51.0%)
2	327 (19.5%)	281 (23.4%)	608 (21.1%)
3	30 (1.8%)	24 (2.0%)	54 (1.9%)
4	2 (0.1%)	0 (0.0%)	2 (0.1%)
X	53 (3.2%)	34 (2.8%)	87 (3.0%)
Missing	45 (2.7%)	72 (6.0%)	117 (4.1%)
Invasive cancer grade, *n* (% of invasive cancer)			
G1	288 (22.0%)	230 (23.4%)	518 (22.6%)
G2	771 (58.9%)	551 (56.2%)	1,322 (57.7%)
G3	135 (10.3%)	155 (15.8%)	290 (12.7%)
X	48 (3.7%)	27 (2.8%)	75 (3.3%)
Missing	66 (5.1%)	18 (1.8%)	84 (3.7%)
Invasive cancer size, *n* (% of invasive cancer)			
≤10 mm	475 (36.3%)	350 (35.7%)	825 (36.0%)
10–20 mm	572 (43.7%)	418 (42.6%)	990 (43.3%)
>20 mm	253 (19.4%)	210 (21.4%)	463 (20.2%)
Missing	8 (0.6%)	3 (0.3%)	11 (0.5%)

‘Missing’ means that the corresponding data entry is empty in the official screening documentation. ‘X’ indicates that physicians tried to determine a value but failed to do so. ‘Other’ invasiveness means that it was unclear whether the cancer was DCIS or invasive and other malignant neoplasms. IQR, interquartile range; UICC, Union for International Cancer Control.

### AI normal triaging, safety net trigger and acceptance rates

AI tagged 56.7% (262,055 of 461,818) of the examinations as normal. This proportion was higher in the AI group (59.4%) than in the control group (53.3%; Table 2) due to an observed reading behavior bias. In the AI group (*n* = 260,739), the safety net was triggered for 3,959 (1.5%) examinations, shown in 2,233 (0.9%) examinations and accepted in 1,077 (0.4%) examinations, leading to 541 (0.2%) recalls and 204 (0.08%) breast cancer diagnoses. Conversely, 8,032 (3.1%) examinations in the AI group underwent further evaluation by the consensus group despite being tagged as normal by AI, resulting in 1,905 (0.7%) recalls, 82 (0.03%) biopsies and 20 (0.008%) subsequent breast cancer diagnoses.

**Table 2:** AI predictions and contributions to the radiologists’ decisions

Conditions	AI group (*n* = 260,739)	Control group (*n* = 201,079)	Overall (*n* = 461,818)
AI prediction			
Normal	154,891 (59.4%)	107,164 (53.3%)	262,055 (56.7%)
Not normal	105,848 (40.6%)	93,915 (46.7%)	199,763 (43.3%)
AI prediction normal and…^a^			
… consensus conference	8,032 (3.1%)	5,454 (2.7%)	13,486 (2.9%)
… recall	1,905 (0.7%)	1,162 (0.6%)	3,067 (0.7%)
… preoperative biopsy	82 (0.03%)	86 (0.04%)	168 (0.04%)
… confirmed cancer	20 (0.008%)	25 (0.012%)	45 (0.010%)
Safety net			
Triggered	3,959 (1.5%)	3,140 (1.6%)	7,099 (1.5%)
Shown	2,233 (0.9%)	NA	NA
Accepted	1,077 (0.4%)	NA	NA
Accepted and had a recall	541 (0.2%)	NA	NA
Accepted and confirmed cancer	204 (0.1%)	NA	NA

NA, not applicable.

^a^The percentages relate the absolute numbers to the total number in the study group (or in the whole study sample for ‘overall’), not the subgroup of mammograms tagged ‘normal’.

### Recall, cancer detection rate and positive predictive values

**Extended Data Fig. 2:**
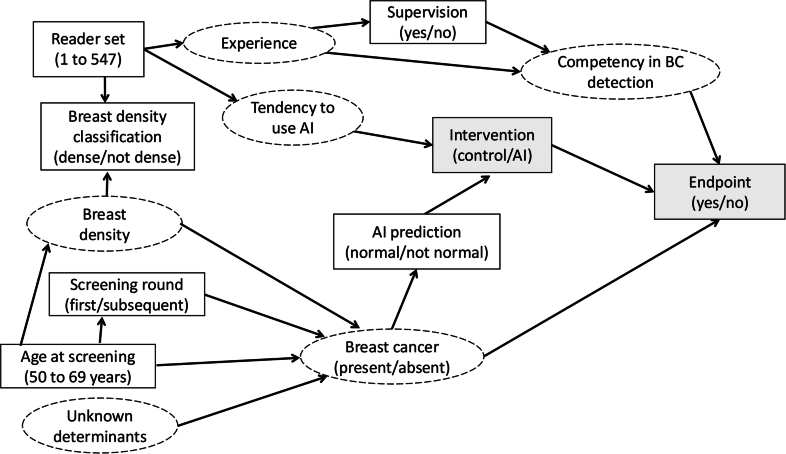
Causal Graph (Directed Acyclic Graph, DAG). Causal graph of assumed relationships between intervention, characteristics of the radiologists and the women screened, and the endpoint (breast cancer detection; recall; consensus conference; biopsy). Before being included in the DAG, possible direct paths between all variables considered were evaluated for plausibility based on theory, domain knowledge and previous empirical evidence. BC: Breast cancer; AI: Artificial Intelligence. Box: observed variable; round and dashed: latent variable.

**Extended Data Fig. 3:**
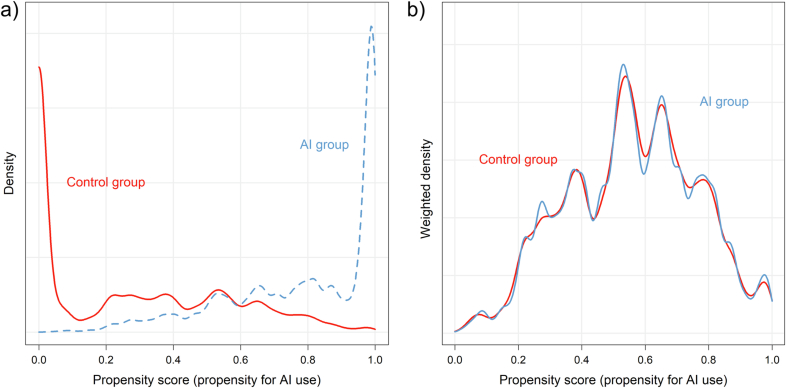
Propensity Scores and Weights. **a**) Distribution of propensity scores (for being in AI group), stratified by being in control group or in AI group. **b**) Extent to which screening exams with a given propensity score (for being in AI group) contribute to the overall sample weight.

We controlled for the identified confounders (reader set and AI prediction; causal graph presented in Extended Data Fig. 2) through overlap weighting based on propensity scores (Extended Data Fig. 3). The model-based breast cancer detection rate (BCDR) per 1,000 women screened was 6.70 for the AI group and 5.70 for the control group. This represents a model-based absolute difference of one additional cancer per 1,000 screened women and a relative increase of 17.6% (95% confidence interval (CI): +5.7%, +30.8%). The BCDR in the AI group was considered noninferior and even statistically superior to that in the control group. The AI group had a lower model-based recall rate (37.4 per 1,000) than the control group (38.3 per 1,000), showing a −2.5% reduction (−6.5%, +1.7%) (Table 3). The positive predictive value (PPV) of recall was 17.9% in the AI group and 14.9% in the control group. The biopsy rate in the AI group was 8.2% higher (−0.4%, +17.6%) than in the control group. Despite this, the AI group demonstrated a statistically significantly higher PPV of biopsy (+9.0% (+2.0%, +16.4%)).

**Table 3:** Model-predicted BCDRs, recall rates, biopsy rates and consensus rates and corresponding differences in the AI and control groups

Variables	Model-based prediction		Model-based difference (95% CI)	
	AI group	Control group	Absolute difference	Percentage difference
BCDR (per 1,000 women screened)	6.7	5.7	1.0 (0.3, 1.7)	17.6% (5.7%, 30.8%)
By invasiveness				
Invasive	5.2	4.8	0.4 (−0.2, 1.0)	7.8% (−4.3%, 21.3%)
DCIS	1.4	0.8	0.6 (0.3, 0.8)	67.6% (29.6%, 116.8%)
Other	0.1	0.04	0.1 (−0.0005, 0.1)	189.6% (−6.6%, 797.7%)
By stage (UICC)				
0	1.4	0.8	0.6 (0.3, 0.8)	67.6% (29.6%, 116.8%)
1	3.3	2.8	0.4 (−0.02, 0.9)	15.4% (−0.9%, 34.4%)
2	1.4	1.5	−0.1 (−0.4, 0.2)	−7.5% (−25.7%, 15.3%)
3	0.1	0.1	−0.04 (−0.1, 0.04)	−34.4% (−71.9%, 53.0%)
4	0.02	≪0.01	0.02 (−0.01, 0.04)	NA
Missing + X	0.5	0.4	0.2 (−0.03, 0.3)	37.8% (−6.4%, 102.9%)
By grade (invasive cancers only)				
1	1.2	0.9	0.3 (0.01, 0.5)	30.0% (0.7%, 67.9%)
2	3.1	2.8	0.3 (−0.2, 0.7)	9.8% (−6.0%, 28.2%)
3	0.6	0.8	−0.2 (−0.5, −0.01)	−28.7% (−48.4%, −1.4%)
Missing + X	0.3	0.3	0.1 (−0.1, 0.2)	24.2% (−23.3%, 101.2%)
By tumor size (invasive cancers only)				
≤10 mm	1.8	1.6	−0.1 (−0.4, 0.2)	8.2% (−11.7%, 32.6%)
10–20 mm	2.4	2.0	0.3 (−0.1, 0.7)	16.1% (−3.0%, 39.1%)
>20 mm	1.1	1.2	−0.1 (−0.4, 0.2)	−9.0% (−29.2%, 17.0%)
Missing	0	0	0.02 (−0.004, 0.04)	NA
Consensus rate (per 1,000 women screened)	112.7	111.1	1.6 (−1.0, 4.2)	1.4% (−0.9%, 3.9%)
Recall rate (per 1,000 women screened)	37.4	38.3	−1.0 (−2.6, 0.6)	−2.5% (−6.5%, 1.7%)
PPV of recall	17.9%	14.9%	3.0 (1.5, 4.6) percentage points	20.5% (6.2%, 32.9%)
Biopsy rate (per 1,000 women screened)	10.4	9.6	0.8 (−0.0, 1.6)	8.2% (−0.4%, 17.6%)
PPV of biopsy	64.5%	59.2%	5.3 (1.3, 9.4) percentage points	9.0% (2.0%, 16.4%)

‘Missing’ means that the corresponding data entry is empty in the official screening documentation. ‘X’ indicates that physicians tried to determine a value but failed to do so. ‘Other’ invasiveness means that it was unclear whether the cancer was DCIS or invasive and other malignant neoplasms.

### Subgroup analyses

Subgroup analyses showed that the BCDR increased in all subgroups by screening round, breast density and age, ranging between +12% and +23% (Table 4). The 95% CIs were completely positive for the subgroups of follow-up screening round, nondense breasts and age 60–69 years.

**Table 4:** Model-predicted BCDRs and recall rates and corresponding differences in the AI and control groups by screening round, breast density and age group

Variables	Model-based BCDR (per 1,000 women screened)			Model-based recall rate (per 1,000 women screened)		
	AI group	Control group	Percentage difference (95% CI)	AI group	Control group	Percentage difference (95% CI)
Screening round						
First	9.3	7.8	19.0% (−4.6%, 48.5%)	94.9	96.5	−1.7% (−7.7%, 4.6%)
Follow-up	6.2	5.2	17.9% (4.4%, 33.1%)	25.8	25.7	0.3% (−5.2%, 6.1%)
Breast density						
Nondense	6.3	5.4	16.6% (1.9%, 33.4%)	32.1	32.9	−2.7% (−7.9%, 2.9%)
Dense	7.4	6.2	18.7% (−0.5%, 41.4%)	47.1	49.0	−3.9% (−9.9%, 2.5%)
Age group						
50–59 years	5.7	5.1	12.1% (−3.6%, 30.5%)	44.3	46.7	−5.0% (−9.8%, −0.2%)
60–69 years	7.9	6.5	22.6% (5.5%, 42.4%)	28.5	27.4	4.1% (−3.6%, 11.9%)

The relative differences in recall rates in the subgroups varied between −5% (age 50–59 years) and +4% (age 60–69 years), but all CIs except for women aged 50–59 years contained zero.

### Sensitivity analyses

We conducted various sensitivity analyses, all of which showed that our analyses were robust to different analytical decisions.

In a model that, in addition to AI prediction and reader set, further adjusted for age, screening round, breast density and supervision in the propensity score model, the BCDR remained unchanged at 17.6% (5.7%, 30.8%). Similarly, in the additionally adjusted model, the PPV of recall and biopsy was 18.3% (−7.3%, 30.5%) and 9.3% (0.5%, 18.8%) higher, respectively, for the AI group than the control group (Extended Data Table 3). The results of the subgroup analyses by age group, screening round and breast density did not change meaningfully following additional adjustments.

Sensitivity analyses in which we adjusted for each reader individually instead of the reader set also provided data similar to the main results: in the AI group, the BCDR was 19.0% (7.4%, 31.8%) higher and the recall rate was −1.5% (−5.4%, 2.6%) lower, indicating that the results were robust to the different parameterization of the reader set variable.

The results were robust toward sampling error, as they remained nearly unchanged when the study sample was varied (bootstrapping and 80% random subset selection, each done 1,000 times): the mean BCDR was 17.6% (5.7%, 30.8%) for bootstrapping and 17.4% (11.4%, 23.8%) for the subset selection.

A propensity score-based alternative to overlap weighting is inverse propensity score weighting with trimming. After applying various trimming thresholds (Extended Data Table 4), the results remained similar.

Another alternative to propensity score weighting as a method for confounder adjustment is stratification. Again, the results of sensitivity analyses including all confounder strata containing a certain minimum sample size (between 0 and 200) in each study group were in line with the main results.

We conducted a placebo intervention analysis to check whether the AI effect observed in the main analysis would vanish (as it should) when there is only a placebo intervention while all assumptions of the model are kept (that is, in the presence of residual confounding due to the reading behavior). As expected, the average model-based difference was minimal (0.8% (−9.9%, 11.6%)), indicating no residual confounding.

### Reading times and workload reduction

**Extended Data Fig. 4:**
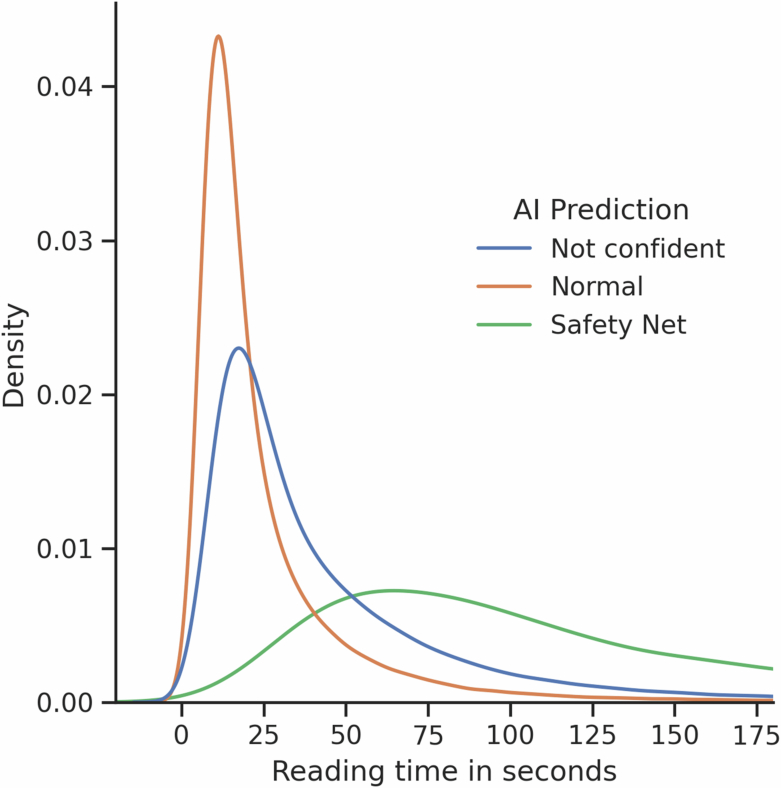
Reading Time. Reading times in the AI group for normal, safety net, and unconfident predictions. On average, examinations that were tagged as normal were read more quickly, with a median reading time of 16 seconds, compared to unclassified examinations, which had a median reading time of 30 seconds, and safety net examinations, which had a median reading time of 99 seconds.

The average reading time per screening examination was measured in the AI group only as it was technically not possible to measure this in the control group. On average, examinations tagged as normal were read more quickly (median reading time, 16 s) than unclassified examinations (median reading time, 30 s) and safety net examinations (median reading time, 99 s). Overall, radiologists spent 43% less time interpreting examinations tagged as normal, with a mean reading time of 39 s for normal examinations compared to 67 s for examinations not tagged as normal (Extended Data Fig. 4).

To evaluate the potential of AI integration to reduce reading workload through automation, we analyzed a fictitious scenario in which the screening examinations triaged as normal by AI were not read by radiologists. Rather, after an AI prediction of ‘normal’, the examination directly received the final classification ‘normal’, and thus, it would not be possible that any breast cancer signs missed by AI were detected by the radiologists, that a recall was made or that a cancer was detected. The analysis of this scenario showed that, when all normal-tagged examinations (56.7%) were automatically classified as normal, the BCDR was still higher and statistically superior by 16.7% (4.9%, 29.9%), the consensus rate was lower by −19.4% (−21.5%, −17.4%), the recall rate was statistically superior and lower by −15.0% (−18.6%, −11.2%), whereas the biopsy rate was higher by 5.8% (−2.7%, 15.0%) in the AI group than in the control group (Table 5).

**Table 5:** Model-predicted BCDRs and recall rates and corresponding differences in the AI and control groups for a fictitious AI automation scenario

Variables	Model-based prediction		Model-based difference (95% CI)	
	AI group	Control group	Absolute difference	Percentage difference
BCDR (per 1,000 women screened)	6.7	5.7	1.0 (0.3, 1.6)	16.7% (4.9%, 29.9%)
Consensus rate (per 1,000 women screened)	89.5	111.1	−21.6 (−24.2, −19.1)	−19.4% (−21.5%, −17.4%)
Recall rate (per 1,000 women screened)	32.6	38.3	−5.7 (−7.3, −4.2)	−15.0% (−18.6%, −11.2%)
Biopsy rate (per 1,000 women screened)	10.1	9.6	0.553 (−0.271, 1.376)	5.8% (−2.7%, 15.0%)

In this fictitious AI automation scenario, screening examinations triaged as normal by AI were not read by radiologists but rather directly classified as normal, resulting in a 56.7% workload reduction for the double-reading system. This analysis used the same model as for the main analysis.

## Discussion

The PRAIM study was embedded within the German breast cancer screening program. To our knowledge, it is the largest study on the effect of integrating AI into mammography screening on the BCDR and recall rate. This study extensively reports AI’s performance in clinically relevant subgroups by screening round, breast density, age, cancer invasiveness, stage, grade and size, with important implications for policy-making. PRAIM included more than 460,000 women, 119 radiologists, 5 different machine vendors and 12 screening sites across Germany. The study did not exclude screening sites, machine vendors, radiologists (for example, based on years of professional experience) or subpopulations of women and allowed for updating the AI algorithms throughout the study, as would occur in a wider rollout. This real-world setting of PRAIM enhances the generalizability of the findings to similar double-reading mammography screening programs. The controlled implementation of AI into the screening process used in the PRAIM study—including onboarding of radiologists on the interpretation of AI recommendations before turning on AI predictions and live monitoring of the AI predictions by the vendor, compliant with postmarketing surveillance regulations—facilitated a safe and responsible rollout of AI^19^.

Our AI approach for mammography screening provided confident normal and confident suspicious predictions (safety net) but no predictions in which it was not confident. The BCDR in the AI group, in which one or both radiologists used the AI-supported viewer to interpret examinations, was 17.6% (5.7%, 30.8%) higher (one additional breast cancer per 1,000 screened women) than in the control group, in which independent standard (human) double reading was done. AI use was also accompanied by a slightly but not statistically significant lower recall rate (−2.5% (−6.5%, 1.7%)).

Our results are in line with earlier published studies. Retrospective studies found a similar or higher BCDR for AI-supported breast cancer screening, suggesting that AI could improve cancer detection by reducing the interval cancer rate and through earlier detection of next-round screen-detected cancers, some of which are retrospectively visible on mammograms from a preceding screening round^12,14,20–22^. Three prospective studies have also reported higher BCDRs with AI-supported screening^15–17^. The MASAI study, a randomized controlled trial, deployed AI to triage examinations for single or double reading in the intervention group and reported a 20% (−0%, 50%) higher BCDR but also increased recall rates from 2.0% (1.9%, 2.2%) to 2.2% (2.0%, 2.3%)^15^. The ScreenTrustCAD study, which had a paired-reader design, showed that the reader replacement approach (AI plus one radiologist) achieved a higher BCDR (4% (0%, 9%)) and a lower recall rate (−4% (−6%, −3%)) than standard independent double reading^16^. Ng et al.^17^ used AI as a third reader to refer examinations for arbitration, which resulted in the detection of additional cancers, most of which were aggressive^17^.

**Extended Data Fig. 5:**
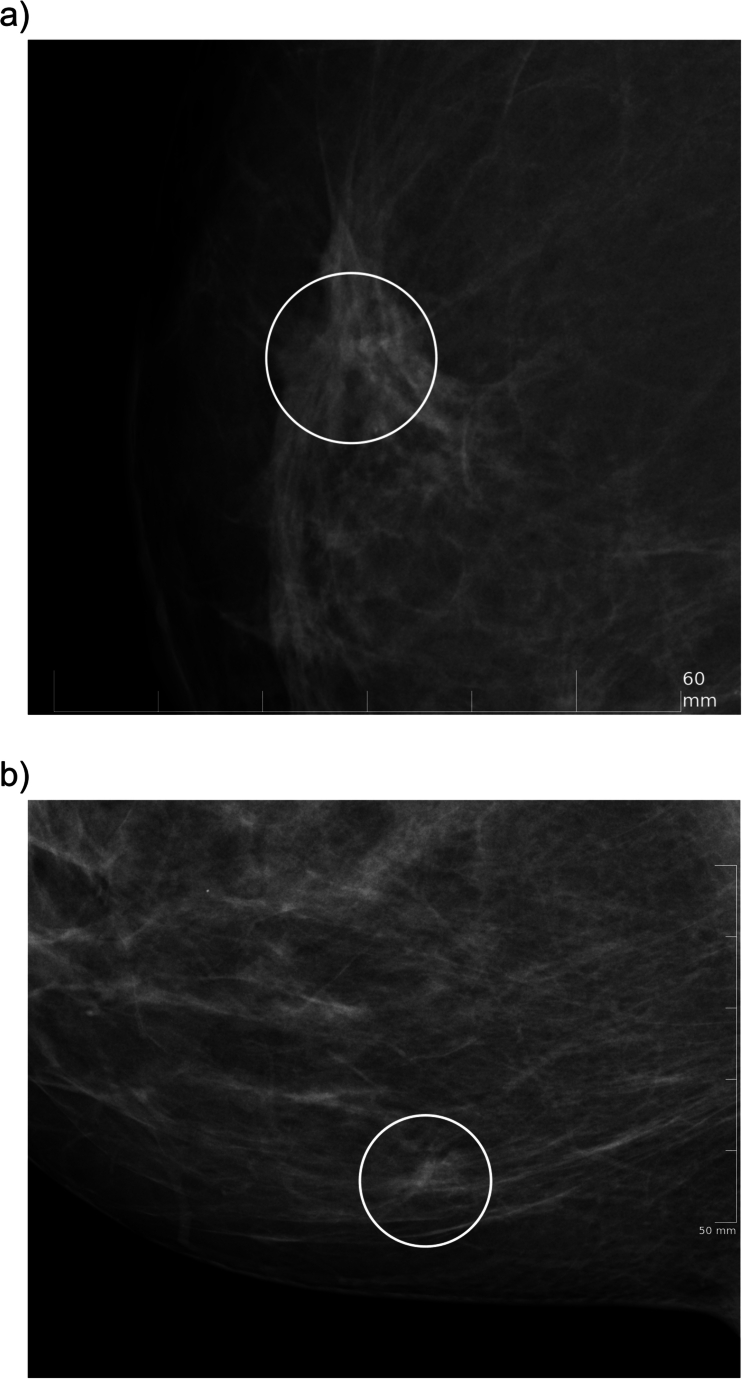
Example Examinations. **a**) This cancer was only diagnosed because of the safety net activation. Neither reader initially saw the invasive carcinoma (BI-RADS 1/2), but both changed their assessment to BI-RADS 4 A after the safety net was displayed. The MLO view of the right breast shows an architectural distortion. Ultrasound during recall identified highly suspicious malignant findings. Histology: Invasive breast cancer, no special type, pT1b (9 mm) pre-therapy, pT1c (19 mm) post-op, N0, M0, G2. **b**) This examination was classified as ‘normal’ by the AI. Both readers used the AI-supported viewer and overruled the AI (BI-RADS 4B and BI-RADS 4 A respectively). The MLO view of the right breast shows a mass. Histology: Invasive breast cancer, no special type, pT1b (9 mm) pre-therapy, ypT1c (12 mm) post-op, N0, M0, G2.

The decision referral approach used in our study allowed for improving the BCDR without increasing the recall rate through a combination of a ‘safety net’ system and ‘normal triaging’. Radiologists using the AI-supported viewer were only alerted and shown suspicious computer-assisted diagnosis marks after they interpreted examinations deemed suspicious by the AI as normal. This approach limits automation bias and reduces false-positive recall rates while leaving the final recall decision to the radiologists (see example cases in Extended Data Fig. 5)^18^. In the AI-supported group, the safety net was triggered 3,959 times and accepted 1,077 times, and 204 breast cancers (61 DCIS, 142 invasive, 1 other) were diagnosed among these safety net-induced reassessments. These breast cancers would have been missed otherwise. Labeling confident negative predictions may exploit automation bias and contribute to reducing the recall rate as radiologists may be less likely to falsely recall examinations tagged as normal by AI. However, radiologists in the AI group also detected 20 cancers among the examinations AI classified as normal.

Although our study and others have shown that the use of AI in breast cancer screening leads to higher BCDRs with comparable recall rates, there are still open questions. First, our study and others suggest that integrating AI into mammography screening might further increase DCIS detection, raising concerns about potential overdiagnosis and subsequent overtreatment. This increase might partially result from the earlier detection of cancers that would otherwise be diagnosed as invasive interval or next-round cancers. It is unclear whether or to what extent the increased BCDR, including the higher detection of DCIS, of AI-supported screening will lead to a lower incidence of interval cancers, better stage distribution of invasive disease and reduced incidence of next-round screen-detected cancers. Knowledge about the importance of higher detection of DCIS and grade 1 cancers on interval cancer rate and stage distribution is crucial but will only become clear after 2–3 years of follow-up. Among others, rejected safety net cases would be an interesting subgroup to help understand the ratio between overdiagnosis and interval or next-round cancers. In the present study, the DCIS detection rate was 0.8 per 1,000 women without AI and 1.4 per 1,000 women with AI, whereas invasive disease was detected in 4.8 per 1,000 women without AI compared to 5.2 per 1,000 women with AI. Once the true extent of overdiagnosis is established in future studies, the potential of AI to detect more DCIS cases that would otherwise not progress to invasive disease should be weighed against the benefit of increased detection of invasive tumors.

Second, it is unknown whether examinations for which the safety net was triggered but rejected by the radiologists represent a correct decision by the reader and thus a critical safety measure to reduce recall and overdiagnosis. Possibly, these cases were missed opportunities to detect even more cancers early and to improve overall program performance further. These questions will be investigated in the 2- to 3-year follow-up analyses. Last, a setting should be defined (for example, by guidelines) and evaluated in which human double reading can be replaced by AI-supported interpretation. This should include a risk–benefit assessment of AI use as well as legal implications.

The integration of AI into screening workflows is expected to alleviate some of the labor shortages experienced by many screening programs. We compared the performance of one or both readers in a double-reading system using AI (deployed in a decision referral approach) to that of both readers not using AI. Therefore, we did not directly assess the extent of reading workload reduction that could be achieved by integrating AI. However, we observed that radiologists in the AI group spent less time interpreting examinations tagged as normal by AI compared to examinations with no confident predictions and examinations with the safety net (Extended Data Fig. 4), thus enabling the radiologists to allocate their time better. In a post hoc analysis assuming that all examinations tagged normal were not read by radiologists and were not forwarded to a consensus conference, we observed a 56.7% reduction in the reading workload. Interestingly, this resulted in a significantly lower recall rate (−15.0% (−18.6%, −11.2%)) while still improving the BCDR by 16.7% (4.9%, 29.9%) (Table 5). This potential workload reduction is comparable to that achieved through the reader replacement strategy used in ScreenTrustCAD and the risk-based triage in MASAI^15,16^.

**Extended Data Fig. 6:**
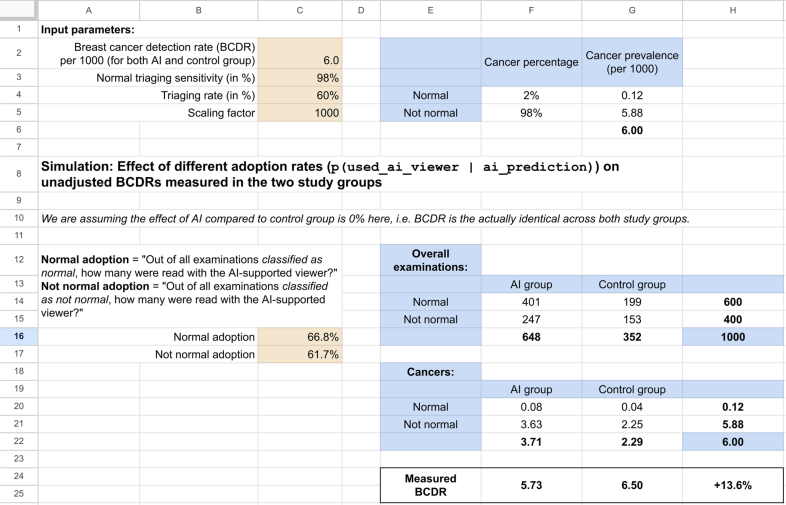
Bias due to self-selection of radiologists to AI or control group. Excel file illustrating the effect of the reading behaviour on measured unadjusted breast cancer detection rate across study groups, even if breast cancer detection rate were identical in both study groups. The figure shows that even a minor adoption rate difference from 61.7% to 66.8% would lead to an unadjusted measured breast cancer detection rate difference of 13.6% (bias). Therefore, it was necessary to control for this reading behavior in the main analysis. The Excel file is available for further analysis (10.5281/zenodo.10822135).

Our study has some limitations. PRAIM is an observational study with no random assignment of screening examinations to the AI-supported and standard-of-care groups. Thus, there was a risk that confounding factors influenced radiologists’ decision to use AI to interpret examinations, which could bias the findings. Indeed, a reader behavior was observed in which some radiologists preferred to read normal-tagged examinations with the AI-supported viewer and to interpret examinations not tagged normal, including those in the safety net, with a standard viewer. Part of this behavior could be due to differences in the functionalities of the available viewers. For example, synchronized zoom (the ability to simultaneously enlarge all four views) is a feature introduced in the AI-supported viewer only during the study, whereas other viewers without AI support typically already have this feature. This reader behavior created a bias (Extended Data Fig. 6) toward a higher breast cancer prevalence within examinations interpreted without AI support. By using propensity scores with overlap weighting (Extended Data Fig. 3), we could overcome this bias on the BCDR as shown in a simulation analysis (‘Statistical methods’ in Methods). Alternative statistical approaches and sensitivity analyses, including additional adjustments for potential confounders, different parameterizations of the reader set variable, placebo intervention analysis, resampling methods and alternative statistical approaches to correct for confounding, consistently demonstrated that our findings are both unbiased and robust.

Our study has several strengths. Besides the high number of participants and the real-world setting, likely leading to more conservative effects, a strength of the study is its prospective design. Retrospective analyses are limited by information bias as the final outcomes for examinations identified as suggestive of cancer only by AI are usually unknown. Our study overcomes this limitation as the AI predictions in the study group were considered by radiologists while making clinical decisions. Another strength of PRAIM is the extensive reporting of subgroup results. They showed noninferior or even statistically superior BCDRs in AI-supported screening across screening rounds, breast densities and ages. Thus, AI can be considered for the full screening population and does not need stratified use. Although not a limitation or a strength, it is worth noting that the data used for this study were collected in the early stages of AI use by radiologists (a learning phase). The interaction behavior between radiologists and AI, and hence the screening program metrics achieved, might change as radiologists become more familiar with using the technology.

In conclusion, our findings substantially add to the growing body of evidence suggesting that AI-supported mammography screening is feasible and safe and can reduce workload. Our study also demonstrates that integrating AI into the screening workflow can improve the BCDR with a similar or even lower recall rate. The important downstream effects of AI-supported screening on overall program performance metrics, including interval cancer rate and stage-at-diagnosis distribution at subsequent screening rounds, are subject to follow-up investigations. Nevertheless, based on the now available evidence on breast cancer detection, recall rates, PPV of biopsy and time savings, urgent efforts should be made to integrate AI-supported mammography into screening guidelines and to promote the widespread adoption of AI in mammography screening programs.

## Methods

### Ethics and data privacy

This research complies with all relevant ethical regulations. Women were informed about the use of AI software at each screening site, and their data were processed according to applicable data privacy rules, including the General Data Protection Regulation. The study protocol was registered in the German Clinical Trials Register (DRKS00027322) and was approved by the ethics committee of the University of Lübeck (22-043), which waived the need for informed consent.

### Procedures

The AI models used in the study are part of a commercially available AI system (Vara). Different deep learning-based AI models were used for normal triaging and the safety net. These models were constructed from a combination of deep convolutional neural networks trained on mammography images. The labels (normal, benign, malignant) for these images were sourced from radiological reports, annotations of findings and biopsy data. The outcomes of ensuing screenings were used to confirm samples labeled ‘normal’. The AI system evaluates the suspiciousness of mammography examination findings by combining per-image scores of incident images and their priors (if available) in an aggregation model. The final score represents the model’s confidence estimate of suspiciousness for a specific case. The models underwent two optimization processes, one aimed at high sensitivity (normal triaging) and the other at high specificity (safety net). Example examinations for AI misclassifying a cancerous case as normal as well as correctly catching a cancer that both radiologists missed through the safety net are shown in Extended Data Fig. 5. The models were trained and validated on a dataset comprising more than 2 million images to identify malignant breast tissue in mammograms. To facilitate the training of the AI models, the radiologists annotated more than 200,000 images with polygons. The models internally output a score between 0 and 1 for each examination, in which 0 means maximally unsuspicious and 1 means maximally suspicious. Thresholds were calibrated based on separate retrospective datasets representative of German screening^18^ and then applied to derive binary decisions (normal versus not normal, safety net versus no safety net) at an average of 60% normal triaging rate and 1.5% safety net trigger rate. For the remaining examinations, neither the ‘normal’ tag shown nor the ‘safety net’ is active. All radiologists who contributed examinations to the AI group were trained as part of the standard medical device onboarding process before they started using the AI-supported viewer. The training covered the functionality of the software, including the interpretation of normal triage flags and safety net alerts.

During the study, the medical device being examined underwent a series of updates, transitioning from version 1.0.5 to 2.6.2 (a total of ten updates). The AI models, which form the basis of the medical device, were updated three times, none of them using data from the study.

Data on the procedure, outcomes, participants and detected cancers were retrieved from the standardized documentation of the official mammography screening program (linked to the data of the AI system) and then transferred in anonymized form to an evaluation database. For analysis, women with missing outcome data (666 women, 0.1%; Fig. 1) or cancellations (610 women, 0.1%) were excluded.

### Outcomes

The primary outcomes were the BCDR and recall rate. A suspicion of breast cancer after mammography was histopathologically confirmed either through preoperative biopsy or surgical biopsy. Examinations of women without detected breast cancer are typically completed within 2 weeks, but the final documentation of a cancer diagnosis and treatment can take several months. Therefore, follow-up time was set to a minimum of 200 days from the date of screening. A recall was defined as a woman being reinvited for further diagnostic examinations. The BCDR and recall rate were further stratified across screening round (first versus follow-up), breast density (American College of Radiology density levels I/II (‘nondense’) versus III/IV (‘dense’)) and age group (50–59 versus 60–69 years).

The secondary outcome measures were the AI predictions (normal or not normal, safety net) and their role in diagnosis. The safety net was considered ‘triggered’ when the score of the AI model exceeded the calibrated threshold for the safety net, ‘shown’ when the reader initially deemed the examination unsuspicious and was shown the warning and localization of the safety net in the AI-supported viewer, and ‘accepted’ when the radiologist consequently changed their assessment. Lastly, the time radiologists spent per examination was assessed in the AI group, stratified by AI prediction.

### Change of analysis plan due to radiologist self-selection

During the data collection period, it was learned through user feedback sessions with radiologists and the AI vendor that the radiologists’ choice to use the AI-supported viewer for the final mammogram report sometimes depended on the initial AI prediction (normal versus not normal), which was already visible in the worklist (Extended Data Fig. 1). The AI-supported viewer also offers a sorted worklist tab where only ‘normal’ examinations are presented one after another for expedited reading.

A common scenario described was that some radiologists tended to read all current examinations tagged as ‘normal’ in the AI-supported viewer, switched their software afterward and then read the remaining examinations in the control group without AI software. A reason for this behavior is that a consensus conference is more convenient when all participating readers used the same viewer for annotations during the initial reads, as this allows the annotations of all readers to be displayed easily during the consensus conference. In contrast, displaying annotations across different viewers does not work flawlessly owing to a lack of software interoperability. Therefore, when a screening examination was not flagged as normal, some readers tended to switch to the other viewer software to ease work with colleagues not using the AI-supported viewer. Additionally, the AI-supported viewer did not support some features helpful for diagnosing suspicious cases (which are more likely among non-normal examinations), such as synchronized zoom (the ability to simultaneously enlarge all four views). We call this observed behavior ‘reading behavior bias’. This practice was not anticipated in the original study setup and introduced severe selection bias (Extended Data Fig. 6). Data illustrating this bias are shared (Code availability statement).

Thus, the original analysis plan had to be adapted.

### Statistical methods

A sample size of 200,000 women per study group was targeted for assessing the noninferiority of AI in terms of the BCDR with the originally planned analysis. Because of the reading behavior bias described above, which was introduced by real-world actions and not anticipated in the original study setup, a severe bias could be present. A simulation study was conducted to (1) identify a statistical method that can successfully correct for this bias when estimating the effect of AI and (2) estimate the expected power for each considered statistical method. Only one of the considered methods—a simple regression model with cancer detection (yes versus no) as the outcome variable, intervention (AI versus control group) as the predictor, a quasi-binomial error distribution, and overlap weighting using propensity scores^23,24^—provided unbiased results and sufficiently high power in the simulation study. The empirical sandwich variance for propensity score weighting estimators was used^23,24^. Propensity scores depended on the reader set assessing the examination and on the AI prediction (normal versus not normal), which were identified as a minimal adjustment set in a causal graph analysis (see below) and were computed using logistic regression. Overlap weighting was used as some propensity scores were rather extreme (Extended Data Fig. 3a), indicating that some reader sets rarely used the AI-supported viewer, whereas some other reader sets usually did. The objective of overlap weighting is to place the highest weight on data in which the propensity for an examination being read with AI (AI group) and without AI (control group) is quite similar and place less weight on data with extreme propensity scores. Extended Data Fig. 3b shows that, after overlap weighting, the largest proportion of the weights was placed on areas of medium propensity scores (that is, readers who contributed reads to both the AI and control groups) and the distribution of the propensity to be in the AI group was the same in both study groups, indicating a balance in the confounders between the groups^23,24^.

For breast cancer detection, AI-supported reading was considered noninferior if the detection rate in the AI group was at most 10% lower than in the control group. More specifically, the lower bound of a two-sided 95% CI of the difference had to be above the threshold of −10%. If noninferiority is successfully demonstrated, sequential testing for superiority is justified^25^. Statistical superiority is demonstrated if the lower bound of the CI exceeds 0. For recall, AI-supported reading was considered noninferior if the recall rate in the AI group was at most 10% higher than in the control group.

Model-based predictions of the BCDR (in total and by invasiveness, stage, grade and tumor size) and the rates of recall (including the PPV), consensus conference and biopsy (including the PPV) in the AI and control groups were derived from the regressions. Subgroup analyses (by screening round, breast density and age group) were conducted by fitting regressions, as described above, to the data restricted to the respective subgroup and by deriving model-based predictions as before.

Sensitivity analyses addressing additional and residual confounding, sampling error and model decisions were performed (‘Results’). All analyses were conducted with R (version 4.1.3) using the packages PSweight (version 1.2.0)^24^, dagitty (version 0.3.1)^26^ and marginaleffects (version 0.18)^27^, as well as Python (version 3.10)^28^ using the package dowhy (version 0.11.1)^29^. All analyses are shared as part of the code (Code availability statement). Further information is also available upon request to the authors.

### Causal graph (directed acyclic graph)

A causal effect of the intervention (use of AI, main predictor) on the endpoints (breast cancer detection, recall, consensus conference, biopsy) was investigated. All direct paths between all variables considered were evaluated for plausibility based on theory, domain knowledge and previous empirical evidence: breast density decreases with age^30^; participation in more screening rounds increases with age; breast cancer risk increases with age^31^; breast density is associated with breast cancer risk^32,33^; breast cancer prevalence is higher among women attending the first screening round^34^; reader variability exists in the classification of breast density categories^35^; AI predictions are associated with the presence or absence of breast cancer^18^; a reading behavior exists whereby AI predictions influence the use of AI-enabled software; radiologists’ personal preferences and attitudes affect their decision to adopt AI^36^; radiologists have varied levels of expertise and competence in interpreting mammography examinations^37^; the involvement of a supervising radiologist introduces a third reader, affecting both cancer detection and recall; and there are unknown factors that may be associated with increased breast cancer risk.

The resulting causal graph is shown in Extended Data Fig. 2. ‘Reader set’ is a confounder according to the backdoor criterion^38^. As it is impossible to adjust for the latent (unobservable) confounder ‘breast cancer (present or absent)’, we adjusted for a proxy (that is, the correlated variable ‘AI prediction’), which was also the direct cause of the observed reading behavior: once radiologists observed the AI prediction in the worklist, they reconsidered which viewer software to use for submitting the report^39^. Therefore, the main analysis model controlled for these two confounders: reader set and AI prediction.

The three variables ‘screening round’, ‘age at screening’ and ‘breast density’ are ancestors of the intervention and outcome, whereas ‘supervision’ is the ancestor of the outcome. Some authors recommend adjusting for all ancestors of the intervention and/or outcome^39^. Therefore, we have also verified that adding the four variables just mentioned forms a valid adjustment set, using the R package dagitty (version 0.3.1)^26^. Results from a sensitivity analysis in which all four variables were additionally adjusted for are provided (Extended Data Table 3). Adjustment was done by adding the covariates to the propensity score model.

Overall, the causal graph implies the 12 (conditional) independences that should hold in the observed data. We tested this using the kernel-based test of statistical independence by Gretton et al.^40^. Indeed, all independence statements hold, showing that the defined causal graph is consistent with the observed data.

### Reporting summary

Further information on research design is available in the Nature Portfolio Reporting Summary linked to this article.

## Online content

Any methods, additional references, Nature Portfolio reporting summaries, source data, extended data, supplementary information, acknowledgements, peer review information; details of author contributions and competing interests; and statements of data and code availability are available at 10.1038/s41591-024-03408-6.

## Supplementary information

Reporting Summary

## Figures and Tables

**Extended Data Table 1:**
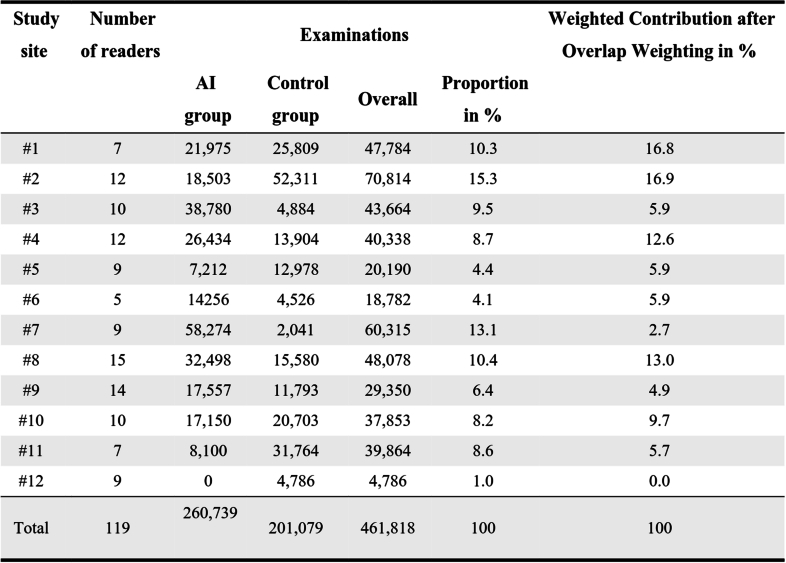
Screening sites participating in PRAIM

**Extended Data Table 2:**
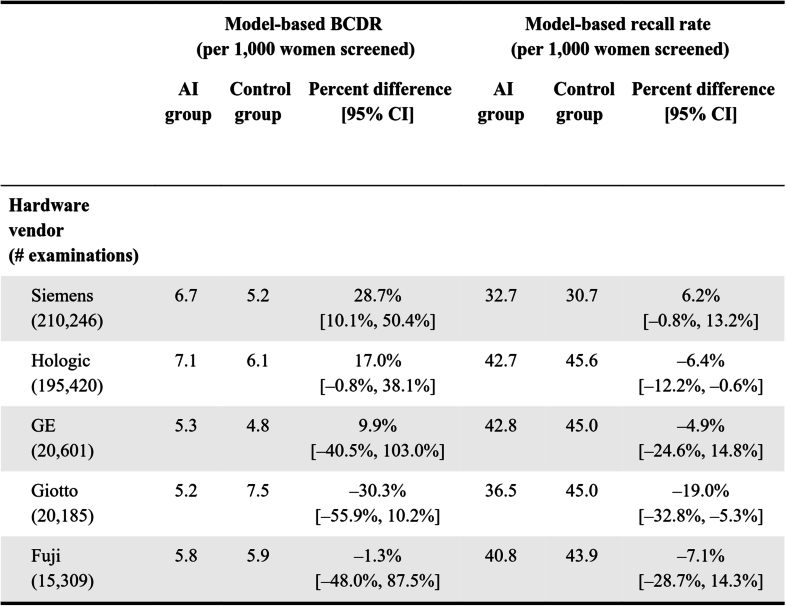
Mammography Hardware Vendors

Model-predicted breast cancer detection and recall rates, and corresponding differences in AI and control group by hardware vendors. For 57 screening examinations, the hardware vendor could not be determined, and therefore those exams are left out of this analysis.

**Extended Data Table 3:**
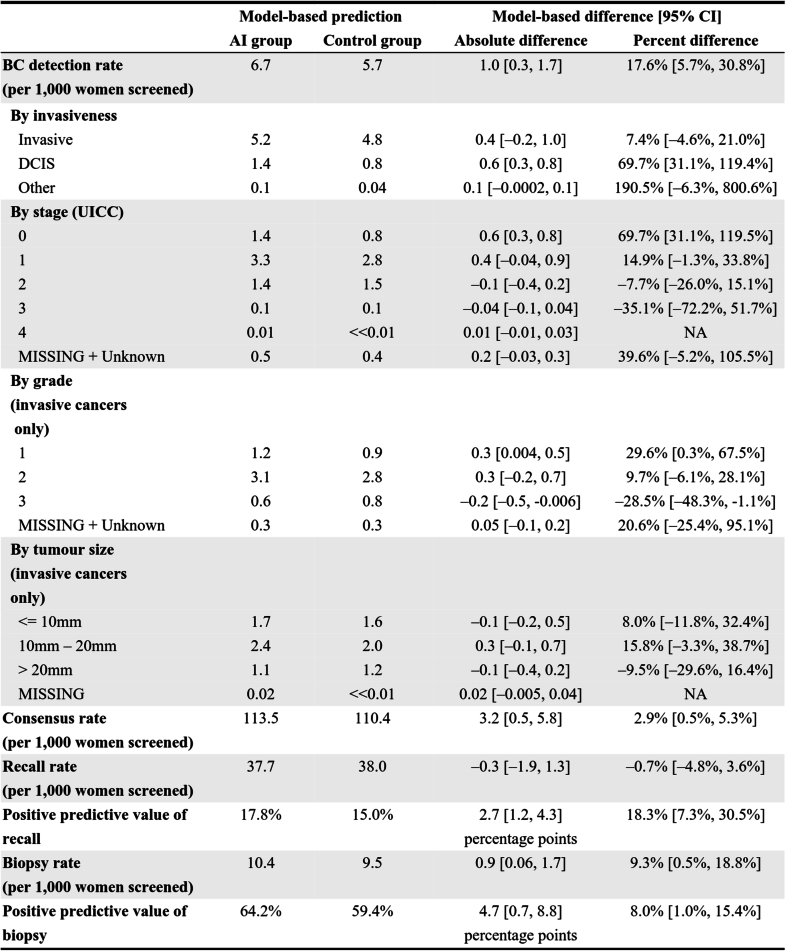
Regression with further covariate adjustment (extended propensity score model)

Model-predicted breast cancer detection rates, recall rates, biopsy rates, consensus rates and corresponding differences in AI and control group (propensity score controlling for reader set and AI prediction and additionally for age, screening round, breast density, and supervision).

**Extended Data Table 4:**
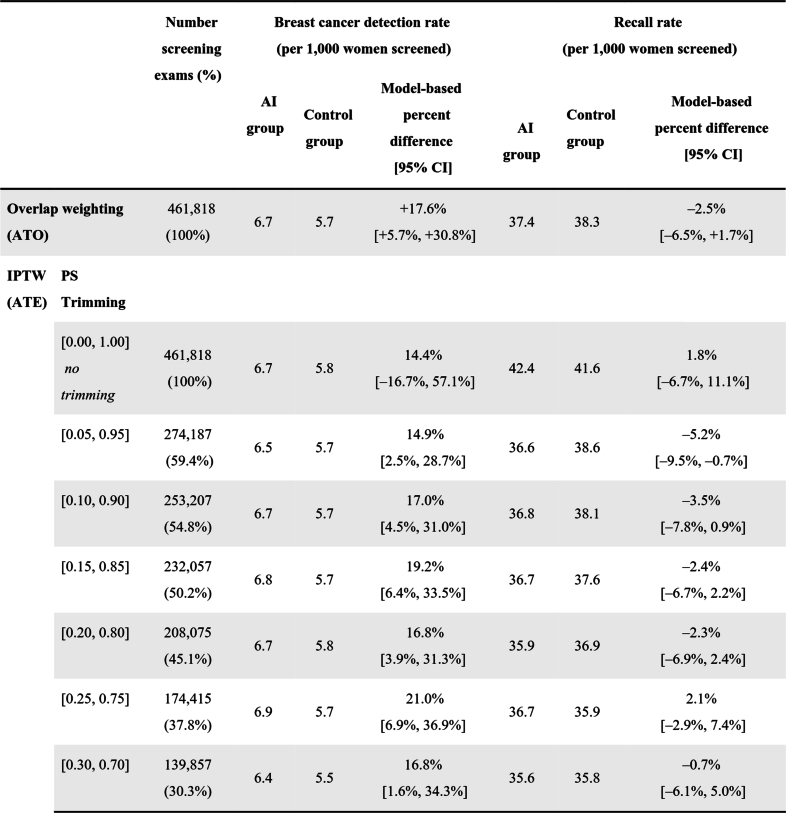
Average Treatment Effect (ATE) via Inverse Propensity Score Weighting (IPTW) and Trimming

Number of included screening examinations and results (breast cancer detection and recall rates) for different propensity score weighting schemes (overlap weighting and IPTW with various trimming cut-offs).

## Data Availability

The study protocol is available at https://research.uni-luebeck.de/en/projects/prospective-multicenter-observational-study-of-an-integrated-ai-s. The anonymized analysis dataset, including individual participant data and a data dictionary defining each field, is available via Dryad at 10.5061/dryad.zs7h44jgn (ref. ^41^). Detailed information on sensitivity analyses and the simulation study is available from nora.eisemann@uksh.de. Requests will be answered within 1 month.

## Code availability

The code and supporting information necessary to reproduce the results are available via Zenodo at 10.5281/zenodo.10822135 (ref. ^42^). The code for training the evaluated AI system is part of a proprietary system and cannot be shared for commercial reasons. However, the AI system is available for external research evaluation collaborations for researchers who provide a methodologically sound proposal. Proposals should be directed to stefan.bunk@vara.ai and will be answered within 1 month.
