# Supplementary material for: Nationwide real-world implementation of AI for cancer detection in population-based mammography screening
Source: Nat Med. 2025 Jan 7;31(3):917–24. doi: 10.1038/s41591-024-03408-6 (PMC11922743; doi:10.1038/s41591-024-03408-6)
Supplement: Supplementary file 1 — Reporting Summary [file 41591_2024_3408_MOESM1_ESM.pdf]

nature portfolio

Corresponding author(s): Alexander Katalinic

Last updated by author(s): Oct 28, 2024

Reporting Summary

Nature Portfolio wishes to improve the reproducibility of the work that we publish. This form provides structure for consistency and transparency in reporting. For further information on Nature Portfolio policies, see our Editorial Policies and the Editorial Policy Checklist.

Statistics

For all statistical analyses, confirm that the following items are present in the figure legend, table legend, main text, or Methods section.

n/a	Confirmed	
☐	☒	The exact sample size (n) for each experimental group/condition, given as a discrete number and unit of measurement
☐	☒	A statement on whether measurements were taken from distinct samples or whether the same sample was measured repeatedly
☐	☒	The statistical test(s) used AND whether they are one- or two-sided<br>*Only common tests should be described solely by name; describe more complex techniques in the Methods section.*
☐	☒	A description of all covariates tested
☐	☒	A description of any assumptions or corrections, such as tests of normality and adjustment for multiple comparisons
☐	☒	A full description of the statistical parameters including central tendency (e.g. means) or other basic estimates (e.g. regression coefficient) AND variation (e.g. standard deviation) or associated estimates of uncertainty (e.g. confidence intervals)
☒	☐	For null hypothesis testing, the test statistic (e.g. F, t, r) with confidence intervals, effect sizes, degrees of freedom and P value noted<br>*Give P values as exact values whenever suitable.*
☒	☐	For Bayesian analysis, information on the choice of priors and Markov chain Monte Carlo settings
☐	☒	For hierarchical and complex designs, identification of the appropriate level for tests and full reporting of outcomes
☒	☐	Estimates of effect sizes (e.g. Cohen's d, Pearson's r), indicating how they were calculated

Our web collection on statistics for biologists contains articles on many of the points above.

Software and code

Policy information about availability of computer code

Data collection	Data was collected via SQL from the anonymized evaluation database.
Data analysis	All analyses were conducted with R 4.1.3 using the packages PSweight 1.2.0, marginaleffects 0.18 and dagitty 0.3.1., as well as Python 3.10 using the package dowhy 0.11.1.<br>The statistical analysis plan is described in the methods section of the manuscript. The original analysis plan had to be updated for reasons detailed there as well. The code is available via Zenodo (https://doi.org/10.5281/zenodo.10822135), see also the corresponding statement in the manuscript.

For manuscripts utilizing custom algorithms or software that are central to the research but not yet described in published literature, software must be made available to editors and reviewers. We strongly encourage code deposition in a community repository (e.g. GitHub). See the Nature Portfolio guidelines for submitting code & software for further information.

Data

Policy information about availability of data

All manuscripts must include a data availability statement. This statement should provide the following information, where applicable:

- Accession codes, unique identifiers, or web links for publicly available datasets
- A description of any restrictions on data availability
- For clinical datasets or third party data, please ensure that the statement adheres to our policy

Data on the procedure, outcomes, participants and detected cancers were retrieved from the standardised documentation of the official German mammography screening programme (MaSc and MammaSoft databases), linked to the data of the AI system, and then transferred in anonymised form to an evaluation database. See the study protocol, available here: https://pure.uni-luebeck.de/ws/portalfiles/portal/62001433/PRAIM_study_protocol_v2.0_final.pdf

The anonymised analysis data set including individual participant data and a data dictionary defining each field is available at Dryad https://datadryad.org/stash/dataset/doi:10.5061/dryad.zs7h44jgn. Detailed information on sensitivity analyses as well as the simulation study are available from nora.eisemann@uksh.de and will be answered within 3 months.
The specific AI models used in this publication are available for external research collaborations for researchers who provide a methodologically sound proposal. Proposals should be directed to stefan.bunk@vara.ai and will be answered within 1 month.

Research involving human participants, their data, or biological material

Policy information about studies with human participants or human data. See also policy information about sex, gender (identity/presentation), and sexual orientation and race, ethnicity and racism.

Reporting on sex and gender	The study deals with breast cancer detection in the context of the German mammography screening. Only those registered as women in the registry office are invited to this programme.
Reporting on race, ethnicity, or other socially relevant groupings	No data on race, ethnicity or other socially relevant groupings are collected in the German mammography screening programme.
Population characteristics	The study population consists of women aged 50 to 69 years, who participate in the German Mammography Screening Programme. Detailed population statistics across both study arms are given in Table 1.
Recruitment	All women attending screening at one of the 12 screening sites in the study time frame were included in the study, no exclusion criteria.
Ethics oversight	The study was approved by the Ethics Committee of the University of Lübeck (22-043). In brief, the study is a non-interventional, observational study with no additional risks for the patients. Only data routinely collected from the mammography screening program linked to the AI system was used. The data were transferred to an anonymized database for analysis. Therefore, the ERB waived the need for informed consent for the study. Further details can be found in the study protocol (https://pure.uni-luebeck.de/ws/portalfiles/portal/62001433/PRAIM_study_protocol_v2.0_final.pdf)

Note that full information on the approval of the study protocol must also be provided in the manuscript.

Field-specific reporting

Please select the one below that is the best fit for your research. If you are not sure, read the appropriate sections before making your selection.

☒ Life sciences ☐ Behavioural & social sciences ☐ Ecological, evolutionary & environmental sciences

For a reference copy of the document with all sections, see nature.com/documents/nr-reporting-summary-flat.pdf

Life sciences study design

All studies must disclose on these points even when the disclosure is negative.

Sample size	A sample size of 200,000 women per study arm was targeted for assessing non-inferiority of AI in breast cancer detection rate (BCDR) with the originally planned analysis. The sample size was based on a power analysis via a simulation study with 10,000 simulation runs and a target of 80% power.<br>After the described necessary change of analysis plan was recognized (see methods section), a simulation study (1,000 simulation runs) was conducted to (1) identify a statistical method that can successfully correct for the described reading behaviour bias when estimating the effect of AI and (2) estimate the expected power for each considered statistical method, given the originally planned study accrual. Only one of the considered methods – a simple regression model with cancer detection (yes/no) as outcome variable, intervention (AI vs. control group) as predictor, a quasibinomial error distribution, and overlap weighting using propensity scores – provided unbiased results and a sufficiently high power (94.6%) in the new simulation study.<br>In the end, data from 461,818 women (260,739 in AI group, 201,079 in control group) were included.
Data exclusions	1303 women were excluded because of technical issues, cancellation of the screening process by the women or unfinished screening process.

Replication	Results can be reproduced given the publicly available data in Dryad and code in Zenodo. NE, HB, and SB checked the correctness of the results. We report several sensitivity analyses in the manuscript and results are shown in the extended data.
Randomization	No randomization was performed (real-world data study).
Blinding	Assignment of study arm was unknown to women and radiographers as it was not yet assigned at the time of image acquisition. No blinding of radiologists was performed/possible.

Reporting for specific materials, systems and methods

We require information from authors about some types of materials, experimental systems and methods used in many studies. Here, indicate whether each material, system or method listed is relevant to your study. If you are not sure if a list item applies to your research, read the appropriate section before selecting a response.

Materials & experimental systems

n/a	Involved in the study	
☒	☐	Antibodies
☒	☐	Eukaryotic cell lines
☒	☐	Palaeontology and archaeology
☒	☐	Animals and other organisms
☐	☒	Clinical data
☒	☐	Dual use research of concern
☒	☐	Plants

Methods

n/a	Involved in the study	
☒	☐	ChIP-seq
☒	☐	Flow cytometry
☒	☐	MRI-based neuroimaging

Clinical data

Policy information about clinical studies

All manuscripts should comply with the ICMJE guidelines for publication of clinical research and a completed CONSORT checklist must be included with all submissions.

Clinical trial registration	German Clinical Trials Register (DRKS00027322) (https://drks.de/search/en/trial/DRKS00027322)
Study protocol	Available here: https://pure.uni-luebeck.de/ws/portalfiles/portal/62001433/PRAIM_study_protocol_v2.0_final.pdf
Data collection	Between July 1, 2021, and February 23, 2023, data from all screened women was collected from 12 screening sites across Germany (Mittelrhein, Niedersachsen-Süd-West, Niedersachsen-Nord, Hannover, Herford/Minden-Lübbecke, Steinfurt, Köln rechtsrheinisch / Leverkusen, Wuppertal / Remscheid / Solingen / Mettmann, Niedersachsen-Mitte, Südwestliches Schleswig-Holstein, Wiesbaden, Niedersachsen-Nordwest).
Outcomes	The primary outcomes were breast cancer detection and recall rate. Breast cancer has to be confirmed either through pre-operative biopsy or surgical biopsy. Recall was defined as a woman being reinvited for further diagnostic examinations. Secondary outcomes were the AI metrics (normal/not normal; safety net) and their role in diagnosis, as well as the screen-reading workload (reading times stratified by AI prediction).

Plants

Seed stocks	n/a
Novel plant genotypes	n/a
Authentication	n/a
